# PKM2 suppresses osteogenesis and facilitates adipogenesis by regulating β-catenin signaling and mitochondrial fusion and fission

**DOI:** 10.18632/aging.102866

**Published:** 2020-02-25

**Authors:** Jiachao Guo, Ranyue Ren, Xudong Yao, Yaping Ye, Kai Sun, Jiamin Lin, Genchun Wang, Fengjing Guo, Jun Xiao, Tao Xu

**Affiliations:** 1 Department of Orthopedics, Tongji Hospital, Tongji Medical College, Huazhong University of Science and Technology, Wuhan, China; 2 Department of Rehabilitation, Tongji Hospital, Tongji Medical College, Huazhong University of Science and Technology, Wuhan, China

**Keywords:** PKM2, osteogenesis, adipogenesis, mitochondrial fusion and fission, β-catenin signaling

## Abstract

Bone marrow mesenchymal stem cells (BMSCs) differentiation dysfunction is a common pathological phenotype of several prevalent metabolic and genetic bone diseases. Pyruvate kinase muscle isoenzyme 2 (PKM2) regulates the last step of glycolysis, and its role in BMSCs differentiation is still unknown. In this study, the influence of PKM2 on osteogenesis and adipogenesis was assessed in vitro and in vivo. We found that DASA-58 (the activator of PKM2) reduced the enzymatic activity of ALP, and inhibited the levels of osteogenic marker genes, especially RUNX2, which is a crucial transcription factor for osteogenesis. Besides, we provided evidence that C3k, an inhibitor of PKM2, caused increase in mitochondrial membrane potential and maintained low levels of ROS, and promoted mitochondrial fusion. Furthermore, after treatment with DASA-58, the level of active β-catenin gradually decreased, which also inhibited the transport of active β-catenin into the nucleus, but C3k obviously promoted its nuclear translocation. As for adipogenesis, PKM2 activation increased the expression of adipogenic related genes and decreased active-β-catenin expression, whereas treatment of C3k had the opposite effect. In addition, C3k significantly attenuated ovariectomy-induced trabecular bone loss in vivo. Our findings helped uncover the molecular mechanisms underlying PKM2 regulation of BMSCs differentiation.

## INTRODUCTION

Bone marrow mesenchymal stem cells (BMSCs) are self-renewable and multipotent stem cells with the capacity to differentiate into adipocytes, osteoblasts and chondrocytes [1]. Several prevalent metabolic and genetic bone diseases, including diabetes, glucocorticoid (GC)-related osteonecrosis, osteoporosis and fibrous dysplasia, share a common pathological phenotype of BMSCs differentiation dysfunction [2–4]. Maintaining the balance of BMSCs differentiation is vital for bone homeostasis. In many cases, factors promoting osteogenesis are recognized as inhibitors for adipogenesis and vice versa [5]. These medical breakthroughs spurred an incredible interest in treating bone loss. Thus, regulating the balance between osteogenesis and adipogenesis can provide appropriate therapeutic targets for preventing or treating insufficient bone formation and excessive bone marrow adipogenesis.

The Wnt/β-catenin signaling is a crucial regulator of BMSCs differentiation [6]. Activation of this signaling can promote BMSCs osteogenic differentiation while suppressing adipogenic differentiation [7]. Wnt ligands bind to Frizzled and low-density lipoprotein receptor-related protein 5/6 (LRP5/6) receptors on the cell membrane, causing inactivation of glycogen synthase kinase-3β (GSK3β) and stabilization of cytoplasmic β-catenin. Then, β-catenin enters the nucleus and integrates with the N-termini of DNA-binding proteins of the T-cell factor/ lymphoid-enhancing factor (Tcf/Lef) family, thereby regulating the expression of downstream target genes such as Runx2 and PPARγ, which respectively are the master transcription factors of osteogenesis and adipogenesis [8, 9].

Mitochondria are essential organelles for eukaryotic cells, they are highly dynamic and constantly undergo fission and fusion [10]. Some researchers have noticed that balanced mitochondrial dynamics and morphology are critical for BMSCs differentiation, and maintaining mitochondrial function is also important for promoting osteogenesis [11]. Through regulating mitochondrial fission and fusion, cells could remove the damaged mitochondria, and restore mitochondrial morphology, membrane potential and function. Mitofusins 1 (Mfn1) and Mitofusins 2 (Mfn2) which belong to membrane-anchored dynamin family stimulate the fusion of outer mitochondrial membranes (OMM), and optic atrophy gene 1 (OPA1) regulates the fusion of inner mitochondrial membranes (IMM). Mitochondria become elongated and coherent during the process of fusion. Fission 1 homolog protein (Fis1) and dynamin-related protein 1 (Drp1) mediate mitochondrial fission, during which mitochondria become shorter and granulated [12, 13]. In addition, Mitochondria are major source of reactive oxygen species (ROS) in mammalian cells [14], and eliminating ROS is crucial for maintaining cellular homeostasis. Increasing in vitro evidence suggests that elevated ROS caused by mitochondrial dysfunction can lead to defect in osteogenic formation. However, low levels of ROS can act as activators that promote osteogenesis [15].

Pyruvate kinase (PK) is the rate-limiting enzyme responsible for the last step of glycolysis, catalyzing dephosphorylation of phosphoenolpyruvate to pyruvate and ATP [16]. Pyruvate kinase muscle isoenzyme 2 (PKM2) is one of the four PK isoforms in mammalian cells, which exists in all cells with high nucleic acid synthesis rates, such as tumor cells, embryonic cells and normal proliferating cells. Pyruvate kinase muscle isoenzyme 1 (PKM1) is an alternative splicing of the same Pkm gene expressed in highly differentiated tissues such as heart, muscle and brain. The remaining isoforms pyruvate kinase liver isoenzyme (PKL) and pyruvate kinase RBC isoenzyme (PKR) are located in the liver and red blood cells, respectively [17]. Specifically speaking, PKM2 exists in two forms and converts between high- and low-activity states. Dimers with low catalytic activity, which are primarily transported to the nucleus and interact with the transcription factors and are responsible for survival and proliferation. Tetramers having high catalytic activity, which exert kinase activity mainly in the cytoplasm [18]. As a specific small molecule activator of PKM2, DASA-58 can transform PKM2 into tetramers, promoting pyruvate kinase activity and remaining in the cytoplasm to enhance glycolysis [19]. In contrast, Compound 3k (C3k), a specific inhibitor of PKM2, has been reported to lock PKM2 into a low activity conformation [20].

The function of PKM2 in osteoarthritis (OA) chondrocytes has been focused by scientists [21], but its effect in bone metabolism is still unclear. In this research, we explored the exact role of PKM2 in osteogenesis and adipogenesis, and provided insights into the mechanisms by which PKM2 regulates β-catenin signaling, mitochondrial morphology and function in differentiation of BMSCs.

## RESULTS

### Effects of DASA-58 and C3k on PKM2 protein expression of BMSCs

**Figure 1:**
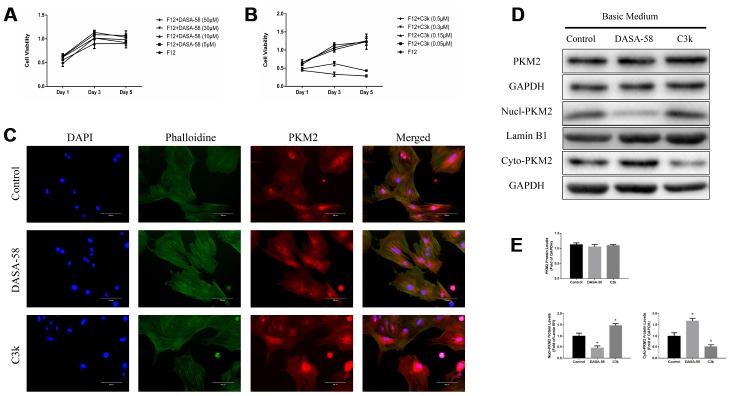
**Verifying changes in PKM2 expression when treated with DASA-58 or C3k.** (**A**, **B**) BMSCs were cultured with different concentration of DASA-58 (0, 5, 10, 30, 50 μM) or C3k (0, 0.05, 0.15, 0.3, 0.5 μM) for 1, 3 and 5 days. BMSCs viability was assessed by CCK-8 assays. (**C**) After 4 days culturing with DASA-58 (30μM) or C3k (0.15 μM), PKM2 immunofluorescence staining on BMSCs was conducted. (**D**, **E**) BMSCs were treated with DASA-58 (30μM) or C3k (0.15 μM) for 7 days. Total proteins, nuclear proteins and cytoplasm proteins were extracted, then total PKM2, nucl-PKM2 and cyto-PKM2 were measured by western blot and quantified. All the experiments have been repeated independently at least 3 times. Data are represented as mean ± SD. *P < 0.05 versus control group.

From the CCK-8 assay it can be seen that 5-50 μM DASA-58 and 0.05-0.15 μM C3k had almost no effect on the viability of BMSCs, and 0.3-0.5 μM C3k suppressed BMSCs viability (Figure 1A, 1B). Figure 1C exhibited that DASA-58 and C3k did not significantly change the total expression of PKM2 in BMSCs, but DASA-58 reduced PKM2 in the nucleus, while C3k significantly increased intranuclear and perinuclear PKM2. For further demonstration, we performed western blot. As shown in Figure 1D, 1E, the total expression of PKM2 was not affected by DASA-58 or C3k, but DASA-58 drastically reduced the protein levels of PKM2 in the nucleus and increased cytoplasmic PKM2, while C3k elevated PKM2 in the nucleus and reduced cytoplasmic PKM2.

### Influence of DASA-58 and C3k on osteogenic differentiation of BMSCs

**Figure 2:**
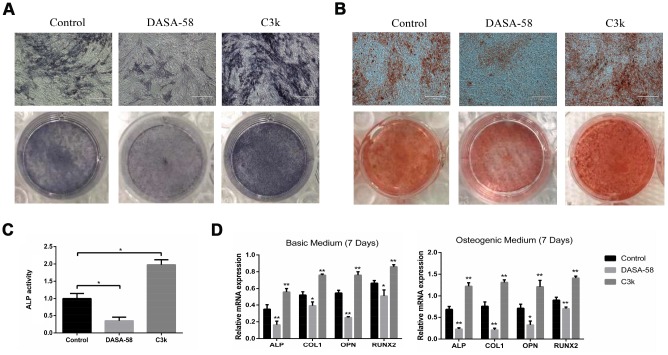
**Impact of PKM2 on osteogenic differentiation of BMSCs.** (**A**) BMSCs were cultured in osteogenic medium with or without DASA-58 (30μM) or C3k (0.15 μM). 7 days later, ALP staining detection was performed. (**B**) Alizarin red staining was performed after 14 days of treatment, and (**C**) ALP activity was measured after 7 days of treatment. (**D**) The relative expression of ALP, COL1, OPN and RUNX2 mRNA levels to GAPDH were detected on 7^th^ day after the induction of basic or osteogenic medium in the presence or absence of DASA-58 (30μM) or C3k (0.15 μM). All the experiments were repeated independently at least 3 times. Data are represented as mean ± SD. *P < 0.05, **P < 0.01 versus control group.

**Figure 3:**
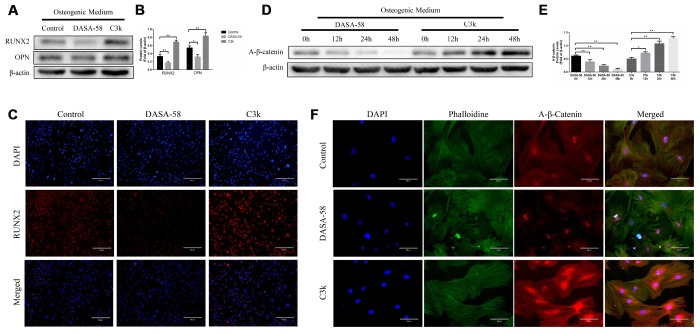
**Inhibition of PKM2 promoted osteogenesis and upregulated the β-catenin signaling pathway.** (**A**–**C**) BMSCs were cultured in osteogenic medium with or without 30 μM DASA-58 or 0.15 μM C3k for 7 days. Protein levels of RUNX2 and OPN were measured by western blot, and immunofluorescence staining for RUNX2 was performed. (**D**, **E**) BMSCs were treated with DASA-58 and C3k for 0h, 12h, 24h and 48h respectively. Protein level of active-β-catenin was detected with western blot. (**F**) BMSCs were cultured with osteogenic medium with or without DASA-58 or C3k for 7 days, then active-β-catenin immunofluorescence staining was performed. All the experiments were repeated independently at least 3 times. Data are represented as mean ± SD. *P < 0.05, **P < 0.01.

Under the premise of culturing in osteogenic medium, the content and activity of ALP in BMSCs treated with DASA-58 for 7 days were significantly decreased, on the contrary C3k increased the content and activity of ALP (Figure 2A, 2C). After the same treatment, alizarin red staining revealed that DASA-58 reduced the formation of calcium nodules in BMSCs under osteogenic culture, while C3k increased the calcium nodules formation (Figure 2B). Figure 2D demonstrated that at the genetic level, cultured in basic medium or osteogenic differentiation medium, BMSCs mRNA levels of osteogenic marker genes ALP, OPN, COL1 and RUNX2 were reduced in various degree on account of DASA-58 treatment, while C3k significantly increased the mRNA expression of these genes. Consistent with these results, western blot evinced that DASA-58 lowered the expression of osteogenic key genes RUNX2 and OPN, and C3k markedly elevated them (Figure 3A, 3B). In addition to this, immunofluorescence staining also verified that the expression of RUNX2 was decreased by DASA-58 and increased by C3k (Figure 3C). As the activation of Wnt/β-catenin signaling is responsible for promoting osteogenesis and inhibiting adipogenesis, we testified by western blot that the expression of active-β-catenin reduced gradually when treated with DASA-58 from 0h to 48h, while C3k made the expression of active-β-catenin increase gradually during this time (Figure 3D, 3E). Figure 3F showed that DASA-58 reduced the expression of active-β-catenin, and the level of nuclear translocation of active-β-catenin was also significantly reduced. C3k increased the expression of active-β-catenin and promoted nuclear translocation of active-β-catenin.

### The regulation effect of DASA-58 and C3k on mitochondrial fission and fusion of BMSCs

**Figure 4:**
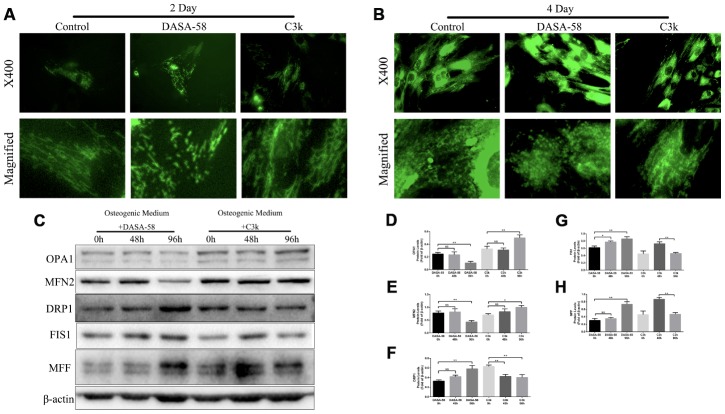
**PKM2 regulated mitochondrial fission and fusion under osteogenic differentiation of BMSCs.** (**A**, **B**) BMSCs were cultured in osteogenic medium and treated with or without 30 μM DASA-58 or 0.15 μM C3k for 2 and 4 days, then stained with mito-tracker green. Images were acquired at 400-fold magnification and further magnified. (**C**–**H**) BMSCs were treated as represented above for 0h, 48h and 96h respectively. Total proteins of OPA1, MFN2, DRP1, FIS1 and MFF were extracted for western blot with the indicated antibodies. Relative protein expression was quantified by Image J and compared to β-actin. All the experiments were repeated independently at least 3 times. Data are represented as mean ± SD. *P < 0.05 and **P < 0.01.

In order to explore the change of mitochondrial fission and fusion caused by DASA-58 and C3k, mitochondrial specific fluorescence staining was performed. Figure 4A, 4B exhibited that on day 2 and day 4, DASA-58 altered mitochondrial shape from wirelike to shorter and granular, which indicated mitochondria were partial to be in a fission process, nevertheless C3k made the mitochondrial appearance thinner and longer showing a fusion characteristic. Afterwards, the results of western blot showed changes in mitochondrial fusion and fission-related genes expressed by BMSCs (Figure 4C–4H). Expression of mitochondrial fusion genes OPA1 and MFN2 were observably lessened after treated by DASA-58 for 96h and promoted when treated by C3k for 96h. Expression of mitochondrial fission genes DRP1, FIS1 and MFF were elevated by DASA-58 after 48 or 96h. DRP1 protein could be lowered by C3k in 48h and 96h, but FIS1 and MFF protein expression showed a trend of increasing first and then decreasing within 0h to 96h.

### Impact of DASA-58 and C3k on mitochondrial membrane potential and intracellular ROS

**Figure 5:**
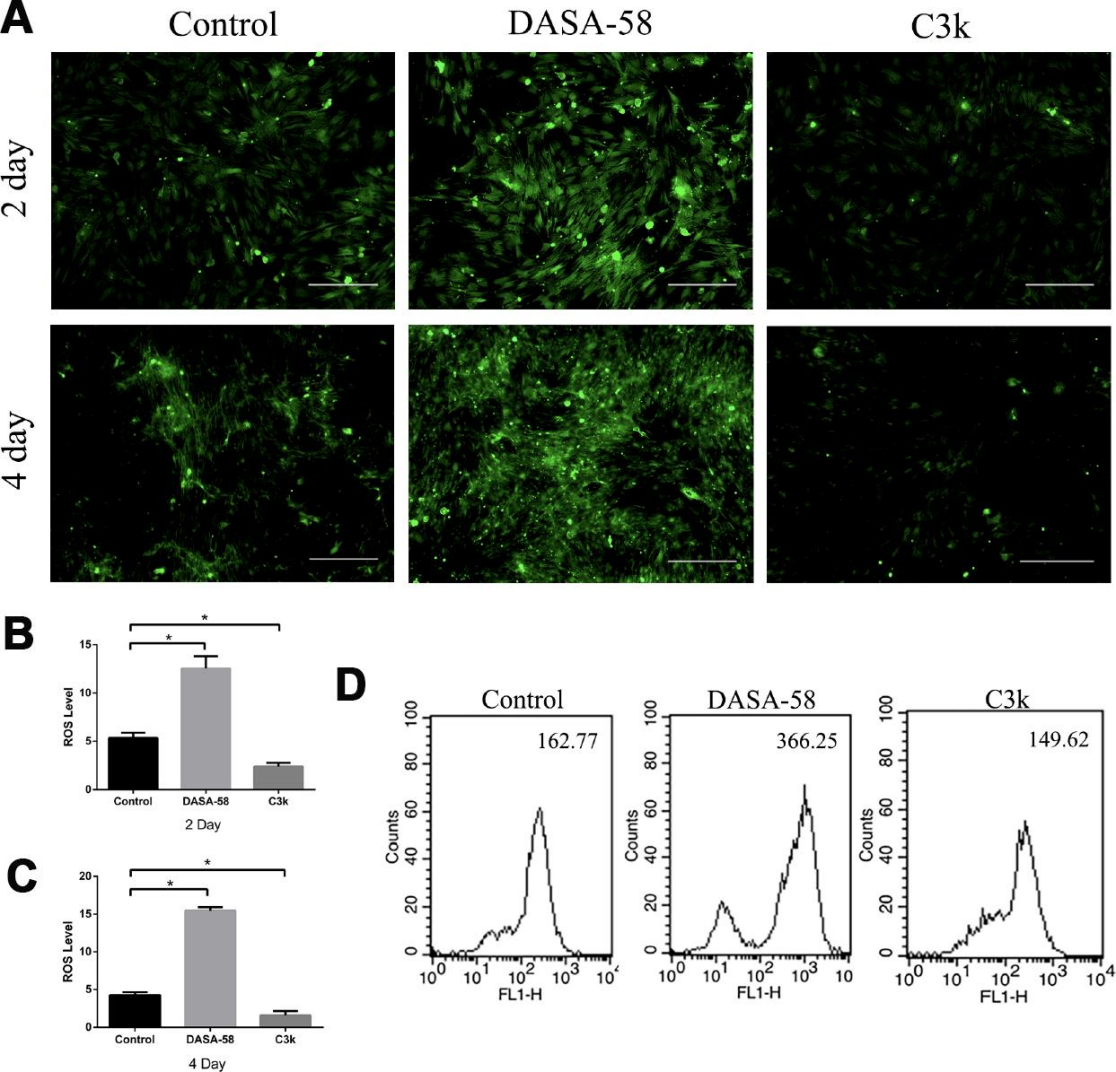
**DASA-58 elevated ROS levels during osteogenesis of BMSCs, while C3k kept it at a low level.** BMSCs were treated with osteogenic medium and treated with or without 30 μM DASA-58 or 0.15 μM C3k for 2 and 4 days, (**A**) then intracellular ROS staining was performed and images were taken, (**B**, **C**) mean fluorescence intensity (MFI) was shown. (**D**) Moreover, after intracellular ROS staining with the fluorescence probe DCFH-DA, flow cytometry was conducted to measure ROS production. All the experiments have been repeated independently at least 3 times. Data are represented as mean ± SD. *P < 0.05 and **P < 0.01 versus the control group.

**Figure 6:**
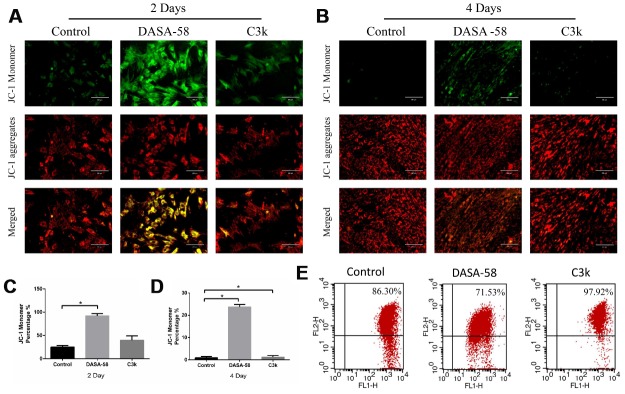
**DASA-58 reduced mitochondrial membrane potential (MMP) of BMSCs during osteogenic differentiation, C3k rose it.** BMSCs were cultured in osteogenic medium added with or without 30 μM DASA-58 or 0.15 μM C3k for 2 and 4 days, (**A**, **B**) then JC-1 staining was performed. Red fluorescence represents JC-1 aggregation in healthy mitochondria, while green fluorescence represents cytosolic JC-1 monomers manifesting MMP collapse. Merged images indicated co-localization of JC-1 aggregates and monomers. (**C**, **D**) JC-1 monomer percentage was shown. (**E**) The flow cytometric analysis was conducted after the cells were stained by JC-1 (FL1 represents JC-1 green and FL2 represents JC-1 red). All the experiments have been repeated independently at least 3 times. Data are represented as mean ± SD. *P < 0.05 and **P < 0.01.

Intracellular ROS and mitochondrial membrane potential both are closely related to the mitochondrial function. Keeping ROS at a low level and maintaining mitochondrial membrane potential at a high level are beneficial to cellular homeostasis and function, including osteogenic differentiation of BMSCs [15]. Figures 5A–5C showed that as DASA-58 was cultured for 2 and 4 days, intracellular ROS increased significantly. As seen in Figure 5D, similar results were shown by flow cytometry, DASA-58 elevated the ROS levels while C3k kept it at a low level. As it is already known, the higher the proportion of JC-1 monomer (green), the lower the mitochondrial membrane potential [22]. DASA-58 increased the percentage of JC-1 monomers, which means that mitochondrial membrane potential decreased on day 2 and day 4, while C3k could maintain it at low levels (Figure 6A–6D). Next flow cytometry showed consistent results that JC-1 red percentage was reduced by DASA-58 and raised by C3k (Figure 6E).

### Effect of DASA-58 and C3k on adipogenic differentiation of BMSCs

**Figure 7:**
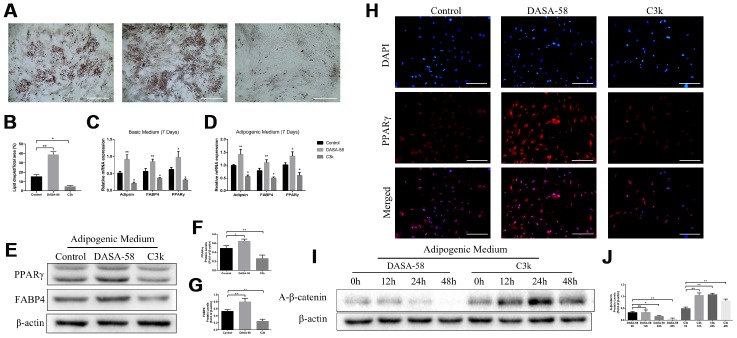
**Effects of PKM2 on adipogenic differentiation of BMSCs.** (**A**, **B**) BMSCs were treated with or without 30 μM DASA-58 or 0.15 μM C3k in adipogenic medium for 14 days, then cells were stained with Oil Red O solution. (**C**, **D**) BMSCs were cultured with basic medium or adipogenic medium supplemented with or without the same concentration of DASA-58 or C3k. 7 days later, mRNA levels of Adipsin, FABP4 and PPARγ were quantified by qRT-PCR. (**E**–**H**) BMSCs were as described above for 7 days, protein levels of PPARγ and FABP4 were detected by western blot, and PPARγ immunofluorescence staining was performed. (**I**, **J**) BMSCs were treated as described above for 0h, 12h, 24h and 48h respectively. Protein expression of active-β-catenin was measured by western blot. All the experiments were repeated at least 3 times independently. Scale bar represents 400 μm. Data are represented as mean ± SD. *P < 0.05 and **P < 0.01.

Results of oil red o staining demonstrated DASA-58 accelerated fat formation upon adipogenic differentiation culturing, while C3k signally inhibited it (Figure 7A, 7B). At the genetic level, mRNA expression of adipogenic marker genes Adipsin, FABP4 and PPARγ were increased by DASA-58 and decreased by C3k under basic medium or adipogenic differentiation medium culturing (Figure 7C, 7D). Similarly, PPARγ and FABP4 protein expression exhibited the same tendency as their mRNA expression (Figure 7E–7G). Immunofluorescence staining again verified that PPARγ expression was evidently arose by DASA-58 while lowered by C3k (Figure 7H). As for Wnt/β-catenin, the key signaling for osteogenesis and adipogenesis, the results of western blot exhibited that active-β-catenin protein expression was decreased after treated with DASA-58 for 24h - 48h under adipogenic induction culture, while the expression of active-β-catenin rose with treatment of C3k for 12h-48h (Figure 7I, 7J).

### C3k prevents OVX-induced bone loss and adipogenesis

**Figure 8:**
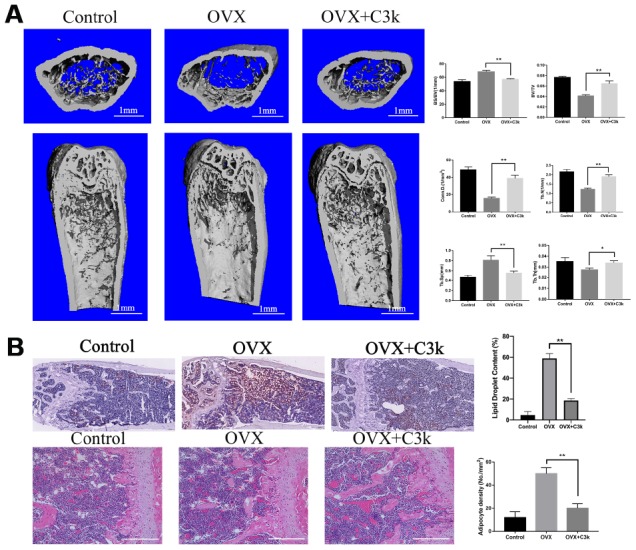
**C3k inhibits bone loss and adipogenesis in OVX mice.** (**A**) Micro-CT images of the distal femoral metaphyseal region from the Control, OVX, and OVX + C3k groups. Histograms represent the trabecular structural parameters of the distal femur: bone surface/trabecular bone volume (BS/BV), trabecular bone volume/tissue volume (BV/TV), connectivity density (Conn.D), trabecular number (Tb.N), trabecular thickness (Tb.Th) and trabecular separation (Tb.Sp). (**B**) H&E staining and Oil Red O staining of distal femoral sections of Control, OVX, and OVX + C3k groups. Scale bar represents 400 μm. Data are presented as means ± SD. n = 10. *P < 0.05 and **P < 0.01.

To evaluate the protective effect of PKM2 inhibition on bone loss in vivo, we used the OVX mice model to imitate osteoporosis in postmenopausal women. Micro-CT was used to analyze the changes in the trabeculae of the distal femur in different model groups. OVX+C3k group showed apparently increase in BV/TV, Conn.D, Tb.N, Tb.Th, while decrease in BS/BV and Tb.Sp when compared with the OVX group. In brief, OVX+C3k group markedly attenuated ovariectomy-induced trabecular bone loss in vivo (Figure 8A). In the selective area of the distal femur from different groups, the number of lipid droplet and adipocytes in OVX+C3k mice significantly decreased compared with OVX mice (Figure 8B).

## DISCUSSION

Osteogenesis and adipogenesis of BMSCs are not independent processes: molecular targets promoting one cell fate suppress the mechanisms stimulating the differentiation of the alternative lineage [5]. Under pathological conditions such as diabetes, osteonecrosis and osteoporosis, BMSCs tend to differentiate into fewer osteoblasts accompanied by more adipocytes [2, 4]. Accordingly, regulating BMSCs differentiation will prove to be valuable for manipulating the above diseases.

PKM2 is the major rate-limiting enzyme in glycolysis, which catalyzes phosphoenolpyruvic acid (PEP) to pyruvic acid and ATP. In cancer research, accumulating evidence shows that PKM2 acts as the key regulator of the Warburg effect, and this protein has been the focus of the global scientists [23]. Besides, PKM2 is a potential target for regulating inflammation, previous study reported that PKM2 could form a transcriptional complex with HIF-1α to activate IL-1β expression [19]. Our data indicated that PKM2 activator and inhibitor have a regulatory effect on adipogenic differentiation and osteogenic differentiation of BMSCs. It is worth noting that PKM2 activator and inhibitor affect PKM2 activity and also change the distribution of PKM2 in the cytoplasm and nucleus. Highly active PKM2 tetramers are mostly distributed in the cytoplasm, while low activity dimers promote PKM2 into the nucleus.

In our study, osteogenesis-related staining showed that activation of PKM2 reduced the formation of calcium nodules and decreased the enzymatic activity and content of ALP. Besides, levels of osteogenic related genes OPN, ALP, COL1 and RUNX2 were reduced by PKM2 activator, especially RUNX2, which is a crucial transcription factor essential for bone formation. There is substantial evidence indicating that mitochondrial morphology and function are modulated during osteogenic differentiation of BMSCs [24]. The dynamic balance of mitochondrial fusion and fission is critical for maintaining mitochondrial membrane potential which regulates mitochondrial function, and mitochondrial dysfunction can show decreased mitochondrial membrane potential and excessive ROS production in mitochondria [25]. Upon osteogenic induction, mitochondrial function is upregulated to meet high energy requirements or to promote other biochemical reactions in the cell [26]. Previous studies demonstrated that expression of the mitochondrial fusion proteins Mfn1 and Mfn2 were increased in the early stages of osteogenesis, along with mitochondrial elongation [27], and that physiologically appropriate ROS levels are essentially required to promote osteogenesis and healthy bone homeostasis [15]. We found that C3k, an inhibitor of PKM2, caused slight increase in mitochondrial membrane potential and maintained low levels of ROS, and that C3k promoted mitochondrial appearance to be thinner and longer, showing mitochondrial fusion characteristics. On the contrary, when treated with the PKM2 activator DASA-58 which visibly inhibits osteogenesis, it was observed that the mitochondrial shape became shorter, the mitochondrial membrane potential was significantly decreased, as well as the intracellular ROS levels were increased. This consistent with the idea that mitochondrial dysfunction and high levels of ROS production will eventually lead to osteogenic inhibition. In short, PKM2 can affect osteogenesis through regulating mitochondrial morphology, mitochondrial membrane potential and intracellular ROS. However, the concrete molecular mechanism of PKM2 affecting mitochondrial fission and fusion and mitochondria function remains to be further studied.

BMSCs mainly rely on glycolysis generating lactate to obtain energy when undifferentiated [28]. This pathway has been proven to provide anabolic and catabolic requirements for maintaining stem cells in a pluripotent state [29]. When not differentiated, BMSCs mitochondria are not yet fully capable of activating oxidative phosphorylation. The oxidative phosphorylation process is activated during the osteogenic differentiation of BMSCs. A highly active oxidative phosphorylation process may be required to meet the high ATP requirements of the extensive biosynthesis of extracellular matrix proteins during osteogenesis [30, 31]. In addition, previous studies have demonstrated that during the osteogenic differentiation of human mesenchymal stem cells (hMSCs), oxidative metabolism of mitochondria is significantly activation, as revealed by upregulation of respiratory enzymes and increased mitochondrial oxygen consumption rate (OCR) [32]. In contrast, during this process, lactate production is reduced and glycolytic activity is drastically reduced. Therefore, metabolic conversion is an important step in the process of osteogenic differentiation of BMSCs [33]. Previous research also confirmed that activation of glycolysis and inhibition of oxidative phosphorylation can inhibit osteogenic differentiation of hMSCs [34].

The connectivity of mitochondria is closely related to the process of cellular oxidative phosphorylation. The fusion and extension of the mitochondrial network transform energy metabolism from glycolysis to mitochondrial respiration (oxidative phosphorylation) [35]. This kind of metabolic transformation caused by mitochondrial fusion is one of the key factors to promote osteogenic differentiation of BMSCs.

In our study, DASA-58, the agonist of PKM2, can make PKM2 to form tetramers and become an active form of the enzyme, promote mitochondrial fission, and inhibit osteogenic differentiation of BMSCs. In contrast, PKM2 inhibitor C3k greatly facilitates osteogenic differentiation of BMSCs. Because C3k caused PKM2 to form an inactive form of the enzyme, which greatly reduces glycolytic capacity, stimulates mitochondrial fusion of BMSCs, and leads to metabolic conversion (from glycolysis to oxidative phosphorylation).

β-catenin plays a vital role in cell adhesion and transcription regulation. The major mechanism for β-catenin transactivation is related to Wnt stimulation and activating mutations of β-catenin partners (such as APC and axin) involved Lef/TCF modulated transcription [36]. Previous studies reported that activation of Wnt/β- catenin signaling inhibits adipogenesis and promotes osteogenesis of BMSCs [5]. After Wnt is activated through the canonical pathway, GSK-3β is inactivated, resulting in β-catenin accumulation in the cytoplasm, nuclear translocation of β-catenin and activation of target genes [37]. Besides, PKM2 could interact with β-catenin. In human glioblastoma multiforme, PKM2 acts as a nuclear coactivator during EGF-induced β-catenin transactivation, and PKM2 binds to c-Srcphosphorylated Y333 of β-catenin [38]. Another research reported that traumatic spinal cord injury induces nuclear translocation of PKM2 to interact with β-catenin and further regulate cyclin protein expression in astrocytes [39]. However, the effect of PKM2 on β-catenin activity has been well proved, whereas the changes in Wnt/β-catenin signaling during PKM2 regulation of osteogenic and adipogenic differentiation are still unclear.

During osteogenic induction, we showed that C3k increased the level of active β-catenin. Interestingly, we testify that expression of active-β-catenin reduced gradually when treated with DASA-58. Besides, DASA-58 also inhibited its translocation into nucleus, whereas C3k obviously promoted this protein nuclear translocation. We hypothesize that nuclear translocation of PKM2 promotes the expression of active-β-catenin in the nucleus. With regard to adipogenesis of BMSCs, increasing evidence shows that the Wnt/β-catenin signaling acts as a negative regulator for adipogenesis. β-catenin modulates its target gene cyclin D1, which inhibits major adipogenic transcription factors such as PPARγ and C/EBP [40]. PKM2 activation increased adipogenesis by inducing the expression of adipogenic related genes, including Adipsin, FABP4 and PPARγ. As for Wnt/β-catenin signaling, active-β-catenin expression was decreased after treated with DASA-58, while the expression of this protein rose with treatment of C3k. In animal experiments, Oil red O staining showed that PKM2 inhibitor significantly reduced lipid droplet content in bone marrow of OVX mice. In addition, traditional sectioning methods have some shortcomings, such as time-consuming, and variation may occur due to differences between laboratories and uneven distribution of bone marrow adipose tissue throughout the medullary canal, so we recommend a new technique that couples histochemical staining of lipid using osmium tetroxide with micro-CT to visualize and quantitate marrow adipose tissue within the medullary canal in three dimensions [41]. Although the link between PKM2 and β-catenin needs to be further clarified in future studies, these data indicate that PKM2 regulates osteogenic and adipogenic differentiation of BMSCs through β-catenin signaling. Moreover, we also found that DASA-58 promoted osteoclastogenesis, while C3k played a significant inhibitory role. However, DASA-58 and C3k have no significant regulatory effect on chondrogenic differentiation of BMSCs (Supplementary Figure 1).

PKM2 plays an essential role in regulating osteogenic and adipogenic differentiation of BMSCs. We found that PKM2 can affect osteogenesis through regulating mitochondrial morphology, mitochondrial membrane potential and intracellular ROS. Moreover, the Wnt/β-catenin pathway is involved in downstream signaling of PKM2 to regulate differentiation of BMSCs and as part of the mechanism by which PKM2 regulates osteogenesis and adipogenesis. We hope that these results can broaden our understanding of PKM2 that could be considered as a new pharmacological target for the therapy of bone diseases.

## MATERIALS AND METHODS

### Reagents and antibodies

DASA-58 (S7928) and Compound 3k (S8616) were purchased from Selleck (Houston, United States) and dissolved in dimethylsulfoxide (DMSO) for use. Rabbit antibodies against MFF (17090-1-AP), OPN (22952-1-AP), FIS1 (10956-1-AP), PPAR-γ (16643-1-AP) and FABP4 (12802-1-AP) were obtained from Proteintech (Rosemont, United States). Rabbit antibodies against PKM2 (#4053), RUNX2 (#12556), active-β-catenin (#19807), OPA1 (#80471), MFN2 (#11925) and DRP1 (#8570) were acquired from Cell Signaling Technology (Boston, United States). Mouse antibody against β-actin (BM3873) was obtained from Boster (Wuhan, China).

### Animal model

Female C57/BL6 mice (12-week old, 22 ± 1g) were obtained from Experimental Animal Center of Tongji Hospital, Huazhong University of Science and Technology (Wuhan, China), fed in the animal care center of Tongji Hospital. Animals were distributed into 3 groups randomly: ovariectomized group (OVX group, n = 10, bilateral ovaries of mice were extirpated via back incision, and from the next day, mice were injected intraperitoneally with vehicle daily), OVX mice treated with C3k group (OVX + C3k group, n = 10, from the following day of operation, intraperitoneal injection of 5mg/kg C3k on mice were conducted daily) and sham-operated group (Control group, n = 10, only explored bilateral ovaries through the back incision). 6 weeks later, the mice were euthanized with excessive pentobarbital to collect femurs, then the femurs were fixed in 4% paraformaldehyde for 2 days. All experiments on animals were authorized by the Ethics Committee on Animal Experimentation of Tongji Medical College, Huazhong University of Science and Technology (Wuhan, China).

### Micro-computed tomography (μ-CT)

After fixation, soft tissue of the femurs were removed and distal femoral cancellous bones were scanned by using micro-computed tomography system (μ-CT50 Scanco Medical, Basserdorf, Switzerland) at 98 μA and 100 kV with a resolution of 10.5 μm. Next we conducted three dimensional reconstruction and analyzed BS/BV, BV/TV, Conn.D., Tb.N, Tb.Sp and Tb.Th in μ-CT system.

### Histologic analysis

All the right femoral heads were decalcified (by using 10% tetrasodium-EDTA aqueous solution), paraffin embedded, and the coronal plane of them were sliced up (4 μm thickness). H&E staining was performed and images were taken by EVOS FL auto cell image system (Life Technologies, United States) and then adipocyte density was quantified.

### Rats BMSCs isolation and culture

BMSCs were isolated from the femurs and tibias of 5 weeks old SD rats [42]. Concisely, euthanized SD rats were sterilized by dipping in 75% ethanol for 15 min, then femurs and tibias of SD rats were separated in a sterile environment and bone marrow were flushed out by using DMED/F12 (Gibco, New York, USA) added 10% Fetal Bovine Serum (FBS) (Gibco) and 1% penicillin/streptomycin (Boster). The culture medium was replaced every 2 days. After reaching 90% clustering, BMSCs were subcultured by digesting with 0.25% trypsin. Third passage of BMSCs was chosen for the following experiments.

### Cell viability assay

Cell Counting Kit-8 (CCK-8, Boster) assay was conducted as described previously [43]. BMSCs were planted in 96-well plates (6 x 10^3^ cells/well) then treated with different concentration of DASA-58 (0, 5, 10, 30, 50 μM) or C3k (0, 0.05, 0.15, 0.3, 0.5 μM) for 1, 3 and 5 days respectively (groups with 0 μM DASA-58 or C3k were treated with equal volume of DMSO). Then BMSCs were incubated with 10% CCK-8 solution at 37°C away from light for 1 hour, then the absorbance at 450 nm wavelength was measured by using ELX800 Absorbance Microplate Reader (Bio-Tec, VI, United States).

### Quantitative real-time reverse transcription-PCR

Quantitative real-time-PCR (qRT-PCR) was used as presented before [44]. Total RNA of BMSCs was extracted by using Total RNA Kit I (Omega Bio-Tek, Norcross, United States), then reverse transcription was performed by Rever Tra Ace qPCR RT Kit (Toyobo, Osaka, Japan). qRT-PCR was conducted with SYBR qPCR Mix (Toyobo, Osaka, Japan) on Bio Rad Q5 instrument (BioRad Laboratories, CA, United States), and the expression of target genes was normalized to *GAPDH*. All experiments were in accordance with the manufacturers’ protocols. Primers were ordered from Tsingke (Beijing, China): (F represents forward; R represents reverse): *PKM2* (F) 5′-TCCCATTCTCTACCGACCTG-3′; (R) 5′-TTCAGTGTGGCTCCCTTCTT-3′; *ALP* (F) 5′-AGGGTGGGTTTCTCTCTTGG-3′;(R) 5′-AGAGAAGGGGTAGGGGAGAG-3′; *COL1* (F) 5′-TCAAGATGGTGGCCGTTACT-3′; (R) 5′-CATCTTGAGGTCACGGCATG-3′; *OPN* (F) 5′-GCGCTCTGTCTCTCTGACCT-3′;(R) 5′-ACCTTATTGCCCTCCTGCTT-3′; *RUNX2* (F) 5′-GGGACCGACACAGCCATATA-3′; (R) 5′-TCTTAGGGTCTCGGAGGGAA-3′; *Adipsin* (F) 5′-CACGTGTGCGGTGGCACCCTG-3′; (R) 5′-CCCCTGCAAGTGTCCCTGCGGT-3′; *Fabp4* (F) 5′-ATGTGCAGAAGTGGGATGGA-3′; (R) 5′-TGCAAATTTCAGTCCAGGGC-3′; *PPARγ* (F) 5′-GGAATCAGCTCTGTGGACCT-3′; (R) 5′-TCAGCTCTTGTGAACGGGAT-3′; *GAPDH* (F) 5′-GGCACAGTCAAGGCTGAGAATG-3′; (R) 5′-ATGGTGGTGAAGACGCCAGTA.

### Western blot analysis

Western Blot analysis was performed as described before [45]. BMSCs were lysed with RIPA Lysis Buffer (Boster) containing 1% PMSF and 1% broad spectrum phosphatase inhibitors (Boster). After centrifugation at 12000 rpm for 30 min, the supernatant was taken and the total protein concentration was detected by BCA (Boster) assay. Equivalent quality of proteins was electrophoresis in 10% SDS-polyacrylamide gel, and transferred to the PVDF membranes (Millipore, United States), then blocked by 5% bovine serum albumin (Boster). Afterwards, the membranes were incubated with respective antibodies overnight at 4°C and incubated for 1 hour with secondary antibodies (Boster) at 25°C. Subsequently, we used enhanced chemiluminescence (Boster) and ChemiDoc^TM^ XRS+ System (Bio-Rad Laboratories, CA, United States) to visualize the proteins.

### Immunofluorescence staining

Immunofluorescence staining was performed as represented before [43]. BMSCs, which were planted in 24-well plates (1 x 10^4^ cells/well) and treated in different ways, were fixed with Immune Staining Fix Solution (Beyotime) for 30 min and then washed by Immunol Staining Wash Buffer (Beyotime). Next, the cells were incubated with QuickBlock™ Blocking Buffer for Immunol Staining (Beyotime) at 25°C for 20 min and then incubated at 4°C with respective antibodies overnight followed by incubating with Cy3 Fluorescent Secondary Antibody (Boster) at 25°C for 1 hour. After the cells were washed, the cells were incubated with Actin-Tracker Green (Beyotime) and DAPI (Boster) for 30 minutes and 5 minutes, respectively. Images were taken by using fluorescence microscope (Evos flauto, Life Technologies, United States).

### ALP staining

BMSCs, cultured in 12-well plates (3 x 10^4^ cells/well) with Mesenchymal Stem Cell Osteogenic Differentiation Medium (Cyagen Biosciences, CA, USA), were treated with or without 30 μM DASA-58 or 0.15 μM C3k for 7 days (groups without DASA-58 or C3k were treated with equal volume of DMSO), then cells were fixed with 4% paraformaldehyde for 15 min. After washing twice with phosphate buffered saline (PBS), the cells were stained by using BCIP/NBT Alkaline Phosphatase Color Development Kit (Beyotime) in line with the manufacturer’s protocol. Images were obtained by using EVOS FL auto cell image system.

### Measurement of ALP activity

BMSCs were cultured with Mesenchymal Stem Cell Osteogenic Differentiation Medium (Cyagen Biosciences) in 6-well plates (2 x 10^5^ cells/well) and treated with or without 30 μM DASA-58 or 0.15 μM C3k for 7 days (groups without DASA-58 or C3k were cultured with equal volume of DMSO). After washing with PBS, BMSCs were lysed with RIPA Lysis Buffer (Boster). After centrifugation, the supernatant was taken and measured by using Alkaline Phosphatase Assay Kit (Beyotime) in accordance with the manufacturer’s protocol, then the absorbance at 405 nm wavelength was tested by using ELX800 absorbance microplate reader (Bio-Tec, VI, United States).

### Alizarin red staining

BMSCs were cultured with Mesenchymal Stem Cell Osteogenic Differentiation Medium (Cyagen Biosciences) in 6-well plates (2 x 10^5^ cells/well), treating with or without 30 μM DASA-58 or 0.15 μM C3k for 21 days (groups without DASA-58 or C3k were treated with equal volume of DMSO). After washing 2 times with PBS, BMSCs were fixed with 4% paraformaldehyde for 15 min. Next, BMSCs were treated with Alizarin Red (Cyagen Biosciences) for 5 min and then were washed 3 times with deionized water. Images were acquired by using EVOS FL auto cell image system.

### Oil Red O staining

BMSCs were cultured with Mesenchymal Stem Cell Adipogenic Differentiation Medium (Cyagen Biosciences) in 6-well plates (2 x 10^5^ cells/well), and treated with or without 30 μM DASA-58 or 0.15 μM C3k for 14 days (groups without DASA-58 or C3k were treated with equal volume of DMSO). Then, BMSCs were fixed with 4% paraformaldehyde for 30 min, and the cells were stained with Oil Red O (Cyagen Biosciences) for 30 min and next washed 3 times. Images were acquired by using EVOS FL auto cell image system. The femurs of mice were fixed with 10% formalin, then rapidly frozen by liquid nitrogen and sectioned. The tissue slices (8μm thick) were dehydrated in anhydrous propylene glycol for 5 minutes, then stained with oil red O solution at room temperature for 30 minutes, next nuclei were stained with Mayer's hematoxylin solution and washed 3 times by water. Observe and photograph the slices with a NanoZoomer S360 (C13220-01, Hamamatsu Japan) and quantify the lipid droplet area by using Image J software.

### Mitochondrial specific fluorescence staining

BMSCs, cultured in 24-well plates (1 x 10^4^ cells/well) with Mesenchymal Stem Cell Osteogenic Differentiation Medium (Cyagen Biosciences), were treated with or without 30 μM DASA-58 or 0.15 μM C3k for 2 and 4 days (groups without DASA-58 or C3k were treated with equal volume of DMSO). After washing, the cells were stained with Mito-Tracker Green (Beyotime) solution for 30min in the dark. The images were taken by using EVOS FL auto cell image system after incubation.

### ROS detection

BMSCs were cultured with Mesenchymal Stem Cell Osteogenic Differentiation Medium (Cyagen Biosciences) in 6-well plates (2 x 10^4^ cells/well), and treated with or without 30 μM DASA-58 or 0.15 μM C3k for 2 and 4 days (groups without DASA-58 or C3k were treated with equal volume of DMSO). Non-fluorescent dichlorodihydrofluorescein (DCFH) can be oxidized by intracellular reactive oxygen species (ROS) to dichlorofluorescein (DCF) with high intensity green fluorescence. According to this theory, we stained the cells by using Reactive Oxygen Species Assay Kit (Beyotime) following the manufacturer’s protocol. The images were acquired by using EVOS FL auto cell image system. For measuring ROS levels, BMSCs were collected and incubated with 10 mM DCFH-DA (Reactive Oxygen Species Assay Kit, proteintech) for 20 min at 37°C away from light. After washing with serum-free medium, mean fluorescence intensity (MFI) was detected by using FACS Calibur flow cytometer (BD, NJ, United States).

### Mitochondrial membrane potential detection

JC-1 is in a state of aggregation and emitted red fluorescence after staining when mitochondrial membrane potential is high. Oppositely, JC-1 presents as monomer and emits green fluorescence after staining when the mitochondrial membrane potential is low [22]. BMSCs were cultured with Mitochondrial Membrane Potential Assay Kit with JC-1 (Beyotime) in accordance with the manufacturer’s protocol and the images were obtained by using EVOS FL auto cell image system. JC-1 owns a potential-dependent accumulation in mitochondria. Mitochondria with normal membrane potential accumulate and produce red fluorescence when high concentrations of JC-1 are aggravated. After treatment, BMSCs were collected and stained with Mitochondrial Membrane Potential Assay Kit with JC-1 (Beyotime), then the results were measured by using FACS Calibur flow cytometer (BD).

### Statistical analysis

One-way analysis of variance was utilized to determine differences among groups more than two by a Tukey test. Intergroup differences between 2 groups were analyzed by Student’s t test. The experiments above were at least 3 times independently, data were presented as the mean ± standard deviation (SD). Statistical significance was indicated by p < 0.05.

## Supplementary Material

Supplementary Materials

Supplementary Figure
