# Supplementary material for: PKM2 suppresses osteogenesis and facilitates adipogenesis by regulating β-catenin signaling and mitochondrial fusion and fission
Source: Aging (Albany NY). 2020 Feb 25;12(4):3976–92. doi: 10.18632/aging.102866 (PMC7066892; doi:10.18632/aging.102866)
Supplement: Supplementary Materials [file aging-12-102866-s001..pdf]

SUPPLEMENTARY MATERIALS

Chondrogenic induction and staining

Chondrogenesis was induced by using Mesenchymal Stem Cell chondrogenic Differentiation Medium (Cyagen Biosciences). BMSCs were grown in the chondrogenic medium for 2 weeks. For Alcian blue staining, BMSCs were washed three times with PBS, and then fixed cells with 4% formaldehyde solution for 30 min. Next, cells were washed with PBS for three times, and stained with Alcian blue for 30 min. Finally, the cells were washed by distilled water for three times. Cells were viewed under EVOS FL auto cell image system. To quantify the proteoglycan, 250 μL 6M guanidine-HCL (Sigma-Aldrich, Oakville, Canada) was added into wells, and then the absorbance at 620 nm was detected for quantitative analysis.

TRAP staining

The femurs and tibias were isolated from 4-6 week-old C57BL/6 mice, the bone marrow cavity was washed with α-MEM medium, and the cells were collected. After 24 hours, floating cells (bone marrow derived macrophages, BMMs) were collected and supplemented with M-CSF, and cultured in a humidified atmosphere of 5% CO2 at 37°C. BMMs (3 × 103 cells/well) were seeded in a 96-well plate, then treated with RANKL and DASA-58 or C3k, and the medium was changed every day. 5 days later, the cells were fixed in 4% paraformaldehyde for 15 min. After washing with PBS, cells were incubated with TRAP staining solution (Sigma-Aldrich, MO, United States) in the dark at 37°C for 60 minutes according to the manufacturer's instructions. Multinuclear TRAP positive cells with nuclei ≥ 3 are defined as osteoclasts.
