# Supplementary material for: PKM2 suppresses osteogenesis and facilitates adipogenesis by regulating β-catenin signaling and mitochondrial fusion and fission
Source: Aging (Albany NY). 2020 Feb 25;12(4):3976–92. doi: 10.18632/aging.102866 (PMC7066892; doi:10.18632/aging.102866)
Supplement: Supplementary Figure [file aging-12-102866-s002..pdf]

SUPPLEMENTARY FIGURE

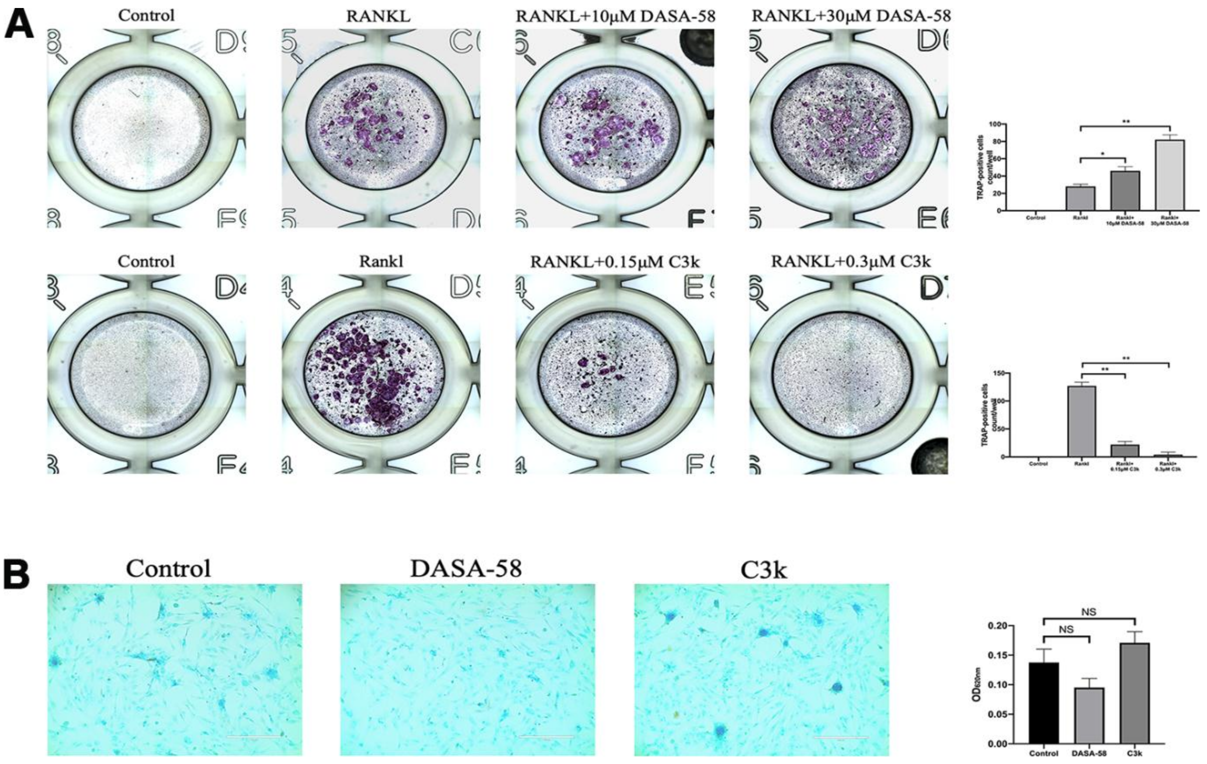

Supplementary Figure 1. Effects of DASA-58 and C3k on osteoclastogenesis and chondrogenesis. (**A**) After seeded in 96-well plates, bone marrow derived macrophages (BMMs) were cultured with α-MEM culture medium and 30 ng/mL macrophage colony stimulating factor (M-CSF). BMMs were treated with 50 ng/mL receptor activator of nuclear factor-κB ligand (RANKL), 50 ng/mL RANKL+10μM DASA-58, 50 ng/mL RANKL+30μM DASA-58, 100 ng/mL RANKL, 100 ng/mL RANKL+0.15μM C3k and 100 ng/mL RANKL+0.3μM C3k respectively. Purple-stained multinucleated giant cells are osteoclasts. As can be seen from the figure, RANKL induced BMMs to form macrophages, while DASA-58 at 10μM and 30μM promotes RANKL-induced osteoclast differentiation, among them, the effect of 30μM DASA-58 is stronger than 10μM DASA-58. C3k of 0.15μM and 0.3μM significantly inhibited RANKL-induced osteoclast formation. In particular, the formation of osteoclasts was hardly seen in the 0.3μM C3k group. (**B**) BMSCs were planted in 24-well plates, then cultured with chondrogenic medium and treated with DASA-58 (30μM) or C3k (0.15μM), respectively. After two weeks, alcian blue staining was performed and the absorbance at 620 nm was detected for quantitative anelysis. The influence of DASA-58 and C3k on chondrogenic differentiation of BMSCs were not statistically significant compared with the Control group.
